# Sentinel Events in Ophthalmology: Experience from Hong Kong

**DOI:** 10.1155/2015/454096

**Published:** 2015-03-02

**Authors:** Shiu Ting Mak

**Affiliations:** ^1^Department of Ophthalmology, United Christian Hospital, 130 Hip Wo Street, Kwun Tong, Kowloon, Hong Kong; ^2^Quality and Safety Office, United Christian Hospital, 130 Hip Wo Street, Kwun Tong, Kowloon, Hong Kong

## Abstract

*Purpose*. To arouse ophthalmologists' awareness in patient safety by reviewing sentinel events in Ophthalmology submitted to a web-based incident reporting system involving all public hospitals in Hong Kong. *Methods*. Sentinel events in Ophthalmology reported from November 2007 to October 2014 were identified and classified into different categories for further presentation and analysis. Key contributing factors attributing to the occurrence of the incidents were described. Suggestions aiming to prevent future occurrence of similar events were made. Relevant literature and case law were discussed. *Results*. Twelve sentinel events were included in this observational case series. They were classified into 4 main categories, namely “wrong eye” (5 cases, 41%), “wrong prescription” (3 cases, 25%), “wrong patient and surgery” (2 cases, 17%), and “retained surgical items” (2 cases, 17%). The key contributing factor leading to the occurrence of the incidents was largely human error. Increased staff awareness and proper time-out procedures were recommended to help prevent occurrence of these errors. *Conclusion*. Sentinel events in Ophthalmology do occur. Many of these incidents were attributed to human error. Surgeon's awareness and willingness to prevent occurrence of sentinel events are warranted.

## 1. Introduction

Since the publication of the landmark report “To Err is Human” by the Institute of Medicine in 2000 [1], studies of errors in medical practice had been increasingly performed. There is no doubt that ensuring patients' safety is a clinician's most fundamental responsibility. To prevent errors from occurring, it is important that clinicians be aware of the potential risks associated with their daily practices. Sharing lessons learned from root cause analysis and policy changes based on root cause analysis review help to reduce harm and adverse events [2].

The Joint Commission on Accreditation of Healthcare Organisations in the United States defined a sentinel event as an unexpected occurrence involving death or serious physical or psychological injury, or the risk thereof. Such events are called “sentinel” because they signal the need for immediate investigation and response [3].

Studies on errors and sentinel events had been widely performed in the fields of medicine, general surgery, and paediatrics. However, there are few such studies in the surgical specialties. As every specialty is unique, each must take responsibility for the study of error within its own domain [4].

This study aimed to arouse ophthalmologists' awareness in patient safety by reviewing sentinel events related to Ophthalmology submitted to a web-based incident reporting system involving all public hospitals in Hong Kong. The submitted incidents were subsequently published in a periodic publication accessible to all healthcare professionals and the public.

## 2. Methods

The Hospital Authority Hong Kong is a statutory body responsible for managing all public hospitals in Hong Kong. The Hospital Authority Advanced Incident Reporting System (AIRS) is a web-based electronic system serving as a tool to support risk management by facilitating the reporting, classification, analysis, and management of incidents. Reportable incidents include any patient safety related incidents, defined as all incidents that are potentially or actually related to patient safety during provision of services. Notification is voluntary and can be done by any staff. In other words, anyone is empowered to report.

The report is submitted via a standardized electronic form. Data collected include patient details, date and details of incident, outcome, and the patient's condition before and after the incident. The information reported to the system remains confidential and can only be assessed by designated staff of the Quality and Safety Office of the respective hospital and the Head Office of the Hospital Authority.

In addition to being an incident notification and reporting system, it allows storage of data, classification of data and contributing factors, supporting incident management, root cause analysis of the individual incident, analysis of data such as number, trend, benchmarking, and risk matrix. These are done by the Quality and Safety Office of the respective hospital and the Head Office. The system also facilitates sharing and learning.

Hospital Authority Risk Alert is a quarterly publication accessible to all healthcare professionals and the public. It is a risk management and communication initiative to further strengthen the reporting and monitoring of adverse incidents in public hospitals. It covers medical incidents that were reported to the Hospital Authority Head Office via the AIRS. The first issue was published in November 2007. To date, 35 issues had been published.

All published issues of the Hospital Authority Risk Alert from November 2007 to October 2014 were reviewed for sentinel events related to Ophthalmology. The events were then classified into different categories for further presentation and analysis. Key contributing factors attributing to the occurrence of the incidents were described. Suggestions aiming to prevent future occurrence of similar events were made. Relevant literature and case law were discussed.

## 3. Results

### 3.1. Case Identification

**Figure 1:**
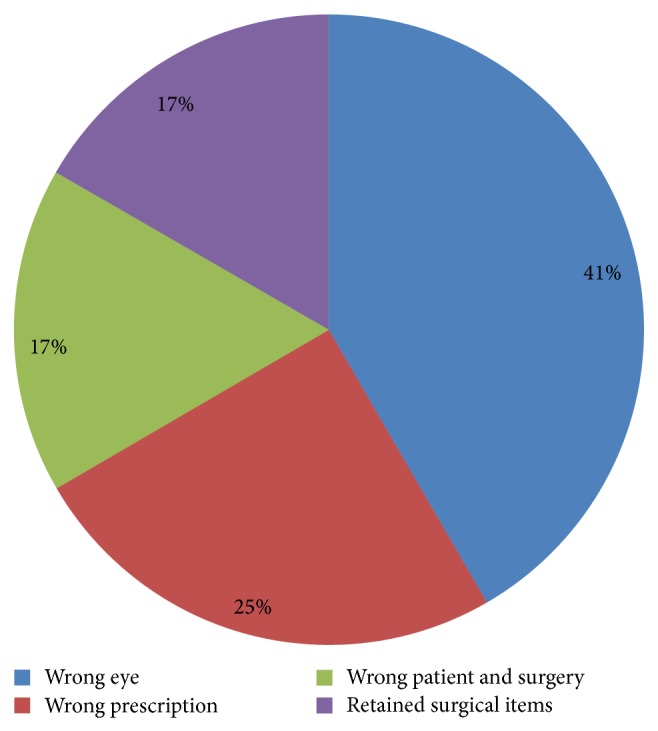
Diagram showing the different categories of sentinel events and their respective percentages.

A total of 12 sentinel events related to Ophthalmology was identified. They were classified into 4 main categories (Figure 1), namely “wrong eye” (5 cases, 41%), “wrong prescription” (3 cases, 25%), “wrong patient and surgery” (2 cases, 17%), and “retained surgical items” (2 cases, 17%).

### 3.2. Wrong Eye

It was identified that this category comprised the largest number of cases. Most cases involved local anaesthetic being injected into the wrong eye. Surprisingly, in almost all cases, the operation site had been marked clearly and correctly, and time-out procedure had been performed by the surgeon and the operating room nurse. Nevertheless, the surgeon subsequently injected the local anaesthetic to the wrong eye despite clear markings. In all cases, the mistakes were immediately identified by other staff in the operating room. Local anaesthetic was then injected to the correct eye with the operation performed. No harm was done to the eye receiving the wrong injection.

**Figure 2:**
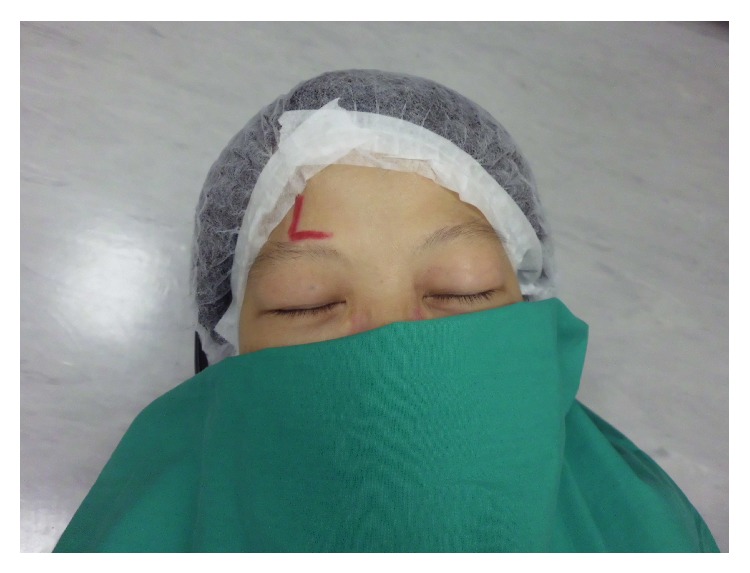
Photograph showing the sign “L” standing for “left” mistakenly marked above the patient's right eyebrow.

The key contributing factor to the error was found to be a long time lapse between time-out procedure and injection of the local anaesthetic, particularly when the injection site was not counter-checked just before the anaesthetic was administered. In two cases, the site markings were covered by the surgical cap the patient was wearing and the headband of the laser machine, respectively. In another case in which a patient was scheduled for left eye operation, the sign “L” standing for “left” was mistakenly marked at the patient's right eye above the right eyebrow (Figure 2).

To avoid this human error, the local anaesthetic should be injected or a surgery should be performed immediately after completing the time-out procedure. Should there be any distractions or extended time lapse between the time-out procedure and the operation, the operating site must be reconfirmed by performing time-out again. Furthermore, the site marking must be correct and clear and should not be blocked or covered by any surgical wraps.

### 3.3. Wrong Prescription

This category involved prescription of wrong intraocular lenses and drugs. An intraocular lens intended for someone else was misplaced in a patient's eye following a swap in the sequence of the operations. The circulating nurse later discovered that a wrong intraocular lens was used. Immediate exchange of intraocular lens had to be performed.

Wrong drug could involve prescribing the wrong medication or at a wrong dosage. Prior to cataract operation, a patient with allergy history to Mydrin P eye drops was given Phenylephrine 2.5% eye drops, which was the active ingredient of Mydrin P. The patient developed mild redness of the eye and mild numbness over the face. The operation has to be postponed. During endoscopic dacryocystorhinostomy for nasolacrimal duct obstruction, the operating surgeon requested for local anaesthetic for injection into the nasal mucosa. Nurses prepared a syringe filled with 1 : 1000 Adrenaline and the syringe was passed to the surgeon. The surgeon injected 1.5 mL of the local anaesthetic into the patient's nasal mucosa and the patient developed tachycardia. Surgery had to be postponed. It was later found out that the intended medication was 2% Lignocaine with 1 : 200,000 Adrenaline.

The key contributing factor of this category of error was the medical staff's failure to check the validity and accuracy of the intended prescription and the staff's wrong subjective assumptions particularly when prescriptions deviated from routine usual practice. From a systems structure point of view, there was lack of an easy reference for commonly used eye medications and dosages.

All prescriptions made must be valid and clear, stating the drug name, dosage, and route clearly. Before application of medications and insertion of intraocular lenses, final cross checking must be done against the correct patient identity and any other relevant information. If order was given verbally, nursing staff should read back the verbal order for confirmation. All staff should be familiar with the dosage of commonly used drugs. Good team work is necessary in counter-checking all prescriptions before administration.

### 3.4. Wrong Patient and Surgery

Rarely, a surgeon might perform surgery on the wrong patient or perform a wrong surgery on a correct patient. Several patients were scheduled for laser therapy in the same session. When the surgeon called Patient A to enter the laser room for treatment, Patient B entered the room instead. The surgeon asked if he was Patient A but he answered yes. Eventually Patient B received the laser treatment intended for Patient A. Similarly, several patients were scheduled for minor eye procedures in the same session. Patient C had dry eyes and was to receive lower eyelid punctal cautery. Three other patients were to undergo syringing and probing of the lacrimal system. On the operation day, the procedure order in Patient C's case notes was missing. The staff assumed that Patient C was also to undergo syringing and probing and it was performed. It was in the subsequent follow-up visit that the doctor found out that Patient C received the wrong procedure.

Failure of the staff in verifying the patient's identity and intended procedure was the key contributing factor of this error. Time-out practice is recommended for all procedures including simple or minor operations. The patient's identity must be checked properly prior to any procedure. If in doubt, staff should recheck the relevant information from the patient's case notes prior to performing the procedure.

### 3.5. Retained Surgical Items

All surgeries require the use of medical instruments and consumables. Sometimes, parts of or entire pieces of these might be retained inside the patient's eye, particularly when ophthalmological surgeries involve the use of small sized instruments and consumables. In a trabeculectomy operation, the surgeon soaked small pieces of sponges with medication and applied the sponges at the operation site. After a few minutes, the surgeon removed the pieces of sponge. When the surgeon examined the patient after the operation, a foreign body was noted at the trabeculectomy site. An operation was subsequently performed, retrieving a small sponge fragment measuring 1 mm in size. During a retinal surgery, a scleral plug was found missing. The operation site was searched but the missing plug could not be located. The patient was sent back to the ward after the operation and X-ray of the orbit was arranged, revealing a shadow compatible with the missing plug. A subsequent operation was performed to remove the retained plug.

Low staff alertness on the possibility of retained surgical items within the surgical field contributed to this error, as staff often mistakenly thought missing surgical items were likely subsequently found outside the surgical field. It is necessary to promote a high index of suspicion among all medical staff for missing surgical item inside the surgical field. Surgeons must check for completeness and integrity of instruments and consumables before closing the operation. Thorough search or assistance from devices such as intraoperative portable X-ray could help to identify any missing surgical item. It would also be beneficial for individual departments to draw up specific safety notes or guidelines for dealing with missing surgical item as future reference.

## 4. Discussion

A considerable proportion of sentinel events identified from this series were related to surgeons performing operations on the wrong eye and wrong patient and carrying out wrong procedures and giving wrong prescriptions. It was suggested that wrong-site, wrong-procedure, and wrong-patient events were likely more common than realized, with little evidence that current prevention practice was adequate. It was estimated that there were 1,300 to 2,700 such incidents annually in the United States involving all specialties [5].

Risk factors for these mistakes include breakdown in communication between the surgeon and the patient and among staff themselves; lack of consistent patient and procedure verification procedures; lack of uniform site-marking procedures; lack of uniform preoperative checklists; incomplete patient assessment; staffing issues; distraction or lack of available information in the operating room; and cultural or language barriers [6]. Much research into the root cause of medical mistakes has found miscommunication to be the key factor [7–9]. Furthermore, the current series found that mistakes were more likely to happen where there were multiple procedures or when unusual patient characteristics caused a break in routine.

Such mistakes may possibly result in a considerably wide range of consequences, including increased patient discomfort and pain, need for extra surgery, increased hospitalization, serious iatrogenic injury, and resultant functional deficit. Fortunately in Ophthalmology, it rarely results in death, though this is possible for other specialties. In many cases, there is permanent harm and it leads to subsequent litigation. In* Green* v.* Wedowee Hosp.* [10], a patient was scheduled to receive cataract operation in his right eye, but the surgeon performed the surgery on the left eye, leaving the patient virtually blind. The patient and his wife sued the surgeon alleging medical malpractice, and the case was settled for US $500,000. In the United States, similar litigation led to an average payment of US $96,032 per claim, with the largest recorded payment being US $9 million [5, 11]. In the United Kingdom, £310,000 was paid out a year by the National Health Service (NHS) to patients who found out that surgeons operated on the wrong part of their body, including being operated on the wrong eye [12].

In the past, marking practices and methods among surgeons varied considerably and ranged from those who always mark to those who never mark or mark only occasionally [13]. In the United States, the Joint Commission on Accreditation of Health Care Organisations has issued guidance on preventing wrong-site surgery, placing a strong emphasis on marking surgical sites before surgery and using a verification checklist [14]. Similarly, in the United Kingdom, the National Patient Safety Agency (NPSA) issued guidance on how to prevent wrong-site surgery in 2005 [15]. However, despite the NPSA issuing the guidance, it had been shown that guidelines were not consistently being implemented by eye surgeons. Noncompliance with side marking was found among 48% of eye surgeons from Scotland, which included both consultants and specialist registrars, bypassing the established multistep process of checks [16]. A similar situation was observed in other specialties. The American Academy of Orthopedic Surgeons had promoted a site-marking policy since 1997 and had publicized it extensively. Despite this, only 70% of orthopedic hand surgeons were aware of the policy, and among them only 45% had changed their practice habits as a result of the new policy [17].

Nevertheless, some surgeons still believed that the most important method of reducing wrong-site surgery is to have a consistent and robust protocol [18]. As a matter of fact, marking the operative site on the patient and time-outs for verbal review of the proposed procedure among all participants are now institutional policy at many facilities, reinforced in some instances by state law or regulation in the United States [19]. A study also showed that using an educational program on wrong-site surgery targeting junior dental staff comprising case-based materials, information feedback from instructor, and clinical guidelines was effective in reducing the incidence of wrong-site tooth extractions [20].

On the other hand, there were concerns that time-out processes planned without consideration of workflow would add more work and ultimately could lead to limited behaviour change [5]. Furthermore, changes to a routine, interruptions, distractions, or too many forms and procedures might detract from safe practice. A series of signatures on the time-out check form might even confer a false sense of security, as it became less likely with each confirmatory signature that an individual would notice or question a mistake [21]. Violations of protocol might occur because multiple checks delayed patient flow so surgeons might deem violations necessary or at least acceptable [6].

Surgeons should bear in mind that whether or not guidelines or protocols exist, the ultimate responsibility for the patient lies with the operating surgeon. The last clear chance of prevention also obviously lies with the operating surgeon. It is therefore of utmost importance that surgeons do not neglect their usual methods for checking the correct site.

During cataract operation, apart from checking patient's identity and side of surgery, time-out procedures should also include checking of the correct intraocular lens before it is implanted. Currently available biometry technology and calculation methods allowed high accuracy in postoperative refractive results [22]. Hence, adverse outcomes following cataract operations were more likely to stem from preventable human errors instead of from technical limitations of biometry [23].

Similarly, retained surgical item is an avoidable error. It was estimated that there were 1,500–2,000 retained surgical item cases a year in the United States [24]. Retained surgical items occurred because of problems with operating room practices and communication, with poor communication being the most common root cause of the error [25]. In particular, each change in shift of staff potentially exposed cases to information loss [26].

Retained surgical item could lead to infection, inflammation, adhesions, fistula or abscess formation, or even sepsis [27]. This created extra pain and discomfort to the patient and further operation is often required to correct the problem. Statistics from the NHS revealed that 199 hospital patients had surgical items unintentionally left inside their body following surgery or other medical treatments in a year. These patients had to spend extra 800 nights in hospital to correct the problem. More than £2 million was paid to the victims, with each of them receiving an average of around £12,500 for the resultant anguish and pain [12].

Surprisingly, it was found out that, in 88% of retained surgical item cases, an instrument count had been performed in the operating room and found to be correct, indicating no missing instruments [28]. It was probable that the operating room staff might have miscounted or reported a correct number of instruments without really doing the count. As in the current series, there were occasionally happenings when a surgical item was recognized as missing as indicated by an incorrect final count; yet the patient was allowed to leave the operating room with the item still inside [24].

It was only under very rare circumstances that surgical items retained inside a patient after surgery could be excusable. Surgeons and nursing staff in charge of the surgery were often held legally responsible for causing the harm and were guilty of malpractice. In the landmark case of* Schorlemer* v.* Reyes* [29], a sponge was left in the patient's abdomen during surgery. Although the two assisting nurses stated that the sole responsibility for removal of surgical sponges rested with them, there was expert testimony that the surgeon was ultimately responsible for ensuring the sponges were completely removed. The surgeon also admitted that the sponges were put in and removed by him and were under his management and control. Thus, the court held that* res ipsa loquitur* (Latin for “the thing itself speaks”) applied and found the surgeon liable.

This kind of error prevention depends on the individual's ability and willingness to use prevention mechanisms. Surgeons had to understand that they bear the final responsibility for ensuring no surgical item was left inside the patient. Even though several systems and technologies are under development to support surgical teams in performing instrument counts to reduce incidents of retained surgical item, additional studies are needed to test the reliability of these new systems [27]. The responsibility of ensuring correct instrument count and complete removal of surgical consumables still rests with the operating surgeon.

Errors and adverse events often occur as a result of system operators deviating from rules, regulations, and standards. The role of leadership in diminishing the frequency and magnitude of adverse events by enforcing rules and standards had been studied. A recent paper had identified leadership and stakeholder partnership important in handling adverse events involving healthcare systems. Leaders should emphasize preparing facilities better before crisis, addressing perceptions of harm, reducing complexity, creating rapid communications with stakeholders, minimizing effects on staff and improving trust, and evaluating and addressing facilities' needs [30].

The data presented in the current study was based on anecdotal incident reports submitted to a web-based electronic system. Leape identified various characteristics of a successful reporting system [31]. The system in the current study is nonpunitive, confidential, and independent and involves expert analysis and is responsive. Nevertheless, it would unavoidably be limited by underreporting. Voluntary reporting was known to underrepresent the total number of adverse events, with medical officer reporting particularly suboptimal [32]. There were various reasons for underreporting. The main reasons included cultural barriers, in particular fear for disciplinary action, shame, and embarrassment, and perceived lack of value in the process. Some might not know what was reportable [33, 34]. Furthermore, low-impact incidents were less likely to be reported, though they were more common than serious adverse events.

It was suggested that there should be legislation to create nonpunitive, confidential reporting systems in order to facilitate dissemination of practice alerts to alert others about error and adverse events [35]. Before this, institutional feedback about reported incidents and system changes to address human errors were essential to improving reporting and reversing the misbelief that only bad doctors made mistakes [33, 34, 36, 37]. Compliance to reporting incidents using reporting systems required dedicated and conscious effort from the entire profession [38]. Frontline staff should understand that collecting patient safety information by them was essential to actively engage the profession in improving patient safety. Root cause analysis of the reported incidents would then allow sharing of lessons learnt to improve patient safety and reduce the occurrence of sentinel events [2].
